# A Randomized Controlled Trial to Measure Spillover Effects of a Combined Water, Sanitation, and Handwashing Intervention in Rural Bangladesh

**DOI:** 10.1093/aje/kwy046

**Published:** 2018-03-27

**Authors:** Jade Benjamin-Chung, Nuhu Amin, Ayse Ercumen, Benjamin F Arnold, Alan E Hubbard, Leanne Unicomb, Mahbubur Rahman, Stephen P Luby, John M Colford

**Affiliations:** 1 School of Public Health, University of California, Berkeley, Berkeley, California; 2 Infectious Disease Division, International Centre for Diarrhoeal Disease Research, Bangladesh, Dhaka, Bangladesh; 3 Infectious Diseases & Geographic Medicine, Stanford University, Stanford, California

**Keywords:** diarrhea, handwashing, herd effects, indirect effects, respiratory illness, soil-transmitted helminths, spillover effects, water and sanitation

## Abstract

Water, sanitation, and handwashing interventions may confer spillover effects on intervention recipients’ neighbors by interrupting pathogen transmission. We measured geographically local spillovers in the Water Quality, Sanitation, and Handwashing (WASH) Benefits Study, a cluster-randomized trial in rural Bangladesh, by comparing outcomes among neighbors of intervention versus those of control participants. Geographically defined clusters were randomly allocated to a compound-level intervention (i.e., chlorinated drinking water, upgraded sanitation, and handwashing promotion) or control arm. From January 2015 to August 2015, in 180 clusters, we enrolled 1,799 neighboring children who were age matched to trial participants who would have been eligible for the study had they been conceived slightly earlier or later. After 28 months of intervention, we quantified fecal indicator bacteria in toy rinse and drinking water samples and measured soil-transmitted helminth infections and caregiver-reported diarrhea and respiratory illness. Neighbors’ characteristics were balanced across arms. Detectable *Escherichia coli* prevalence in tubewell samples was lower for intervention participants’ neighbors than control participants’ (prevalence ratio = 0.83; 95% confidence interval: 0.73, 0.95). Fecal indicator bacteria prevalence did not differ between arms for other environmental samples. Prevalence was similar in neighbors of intervention participants versus those of control participants for soil-transmitted helminth infection, diarrhea, and respiratory illness. A compound-level water, sanitation, and handwashing intervention reduced neighbors’ tubewell water contamination but did not affect neighboring children’s health.

Improvements in household water quality, sanitation, and handwashing practices (WSH) may reduce the risk of diarrhea (1), soil-transmitted helminth (STH) infection (2), and respiratory illness (3, 4). WSH interventions may also reduce illness among neighbors through “spillover effects” (5) (a.k.a., herd effects (6–8) or indirect effects (9)) resulting from 1) reduced fecal contamination in the environment surrounding intervention recipients; 2) reduced pathogen transmission from intervention recipients to neighbors, resulting from recipients’ lower disease burden due to intervention; or 3) adoption of promoted health behaviors by neighbors. If WSH interventions reduce illness among recipients and other individuals, estimates that ignore spillover effects would underestimate the full effect of WSH interventions.

There is a rich literature on herd effects of vaccines (5, 7). The literature on spillover effects for other infectious disease interventions, such as school-based deworming (10) and insecticide-treated nets (11), is growing (5). Whereas WSH interventions’ direct effects on recipients have been measured in many empirical studies (1–3), spillover effects of WSH have been measured in few studies (12–19); observational studies have been used to measure spillovers, so spillover estimates in the studies may have been susceptible to bias if there were systematic differences between individuals in close proximity to the intervention and individuals serving as controls.

We measured spillover effects of a compound-level, combined WSH intervention in an existing, large, rigorously designed trial: the Water Quality, Sanitation, and Handwashing (WASH) Benefits Study in Bangladesh (20). We measured whether compounds neighboring WASH Benefits intervention recipients had lower environmental contamination and whether the children in these compounds had a lower prevalence of STH, diarrhea, and respiratory illness compared with children of neighboring control participants.

## METHODS

### Randomization

We performed a cluster-randomized trial that built on the WASH Benefits Study (20, 21), which was conducted in the Gazipur, Mymensingh, Tangail, and Kishoreganj districts of central Bangladesh. These areas were selected because they had low groundwater levels of arsenic and iron (to avoid interference with chlorine water treatment) and no other WSH or nutrition programs. In the WASH Benefits Study, clusters were randomly assigned 1) drinking water treatment and safe storage, 2) sanitation, 3) handwashing, 4) combined WSH, 5) nutrition, 6) combined nutrition and WSH, and 7) no intervention (control) (21). WASH Benefits Study investigators randomized treatment within geographic blocks containing adjacent clusters. A random-number generator was used to randomly assign participants to treatment or control arms of the study within groups of geographically contiguous clusters. Clusters were separated by at least 1 km to reduce the risk of between-cluster spillovers resulting from reductions in disease transmission or the adoption of interventions in the control group. No evidence of spillovers from the intervention group to the control group was found (20).

**Figure 1.**
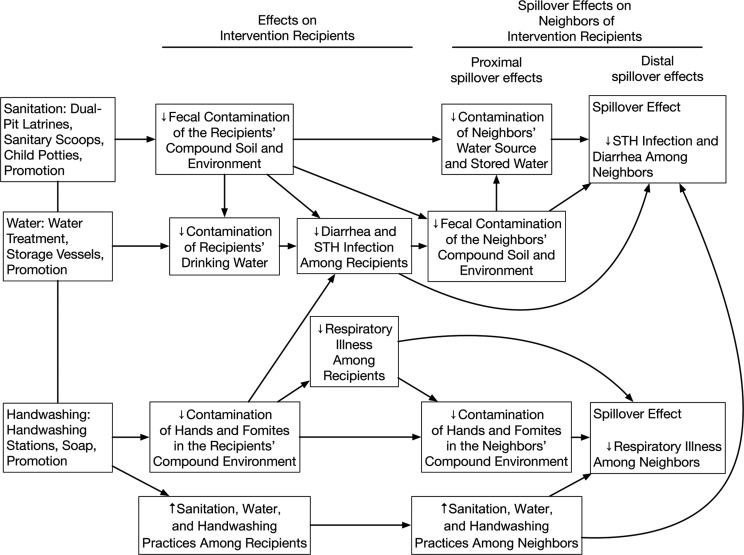
Theoretical model for spillover effects of a compound-level combined water, sanitation, and handwashing intervention in the Water Quality, Sanitation, and Handwashing (WASH) Benefits Study, January 2015–August 2015. Contamination of neighbors’ water source and stored water was measured by enumerating fecal indicator bacteria in drinking-water samples. Fecal contamination of the neighbors’ compound and environment was measured by counting synanthropic flies captured near cooking areas and latrines. Contamination of hands in the neighbors’ compound environment was measured by observing caregiver’s and children’s hand cleanliness. Upward arrows indicate increases; downward arrows indicate decreases. STH, soil-transmitted helminth.

We measured geographically local spillovers among neighbors of trial participants in 90 control clusters and 90 combined WSH intervention clusters in the WASH Benefits Study from January 2015 to August 2015. To measure spillovers, we focused on the combined WSH intervention because we hypothesized that of all intervention packages in the trial, the combined WSH intervention was most likely to produce spillover effects (Figure 1). To coordinate data collection efforts and maximize comparability with the main trial, we selected control clusters where, in the main trial, researchers planned to collect environmental samples. Because interventions included visible hardware, neither the outcome measurement team nor study subjects were masked to intervention assignment.

### Participants

**Figure 2.**
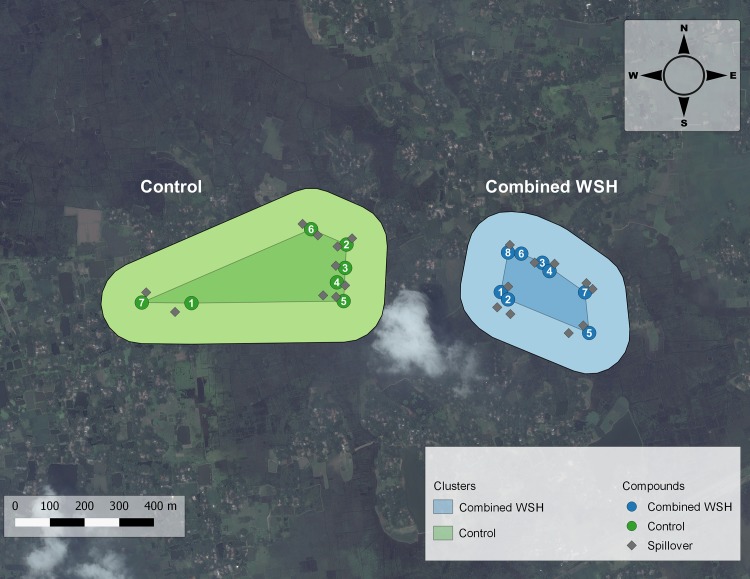
Design of Water Quality, Sanitation, and Handwashing (WASH) Benefits Study, January 2015–August 2015. This figure depicts the study design in 2 clusters: 1 assigned to the combined WSH intervention and the other assigned to control. Each cluster was separated by a buffer zone of at least 1 km to minimize the chance of spillovers between clusters. The numbered circles denote the compounds enrolled in the WASH Benefits Study. The gray diamonds denote the neighboring compounds enrolled in the spillover study. The boundaries of each cluster were not formally defined in the WASH Benefits Study. In this figure, the darker-shaded center of each cluster is the polygon formed by linking the outermost compounds in each cluster, and the lighter-shaded section is the periphery around this polygon. We restricted enrollment to the compounds within this periphery to ensure that the 1 km buffer zone was maintained in this study. WSH, water, sanitation, handwashing.

In rural Bangladesh, families typically live in clusters of households with a common courtyard. Compounds were eligible for the WASH Benefits Study if a pregnant woman resided there at the time of study enrollment who intended to stay in her village during the follow-up period. A birth cohort of “index” children (in utero at enrollment) of enrolled mothers was followed during the trial. After 24 months of intervention, there were 6.4 study compounds per cluster, on average, and these compounds typically composed less than 10% of compounds located within the cluster boundaries. To measure spillovers, we enrolled compounds neighboring those in the WASH Benefits Study in intervention and control clusters concurrent with primary outcome measurement in the main trial. Neighbors were eligible if a child 0–59 months old at the time of spillover study enrollment (just younger or older than the index child cohort) resided there and if they were within 120 steps (2 minutes’ walking time) of a WASH Benefits Study compound (Figure 2). We excluded children enrolled in the WASH Benefits Study and children in compounds that shared a courtyard, latrine, or handwashing station with a WASH Benefits Study compound. Within each cluster, there were typically 6–8 WASH Benefits Study compounds. For the spillover study, fieldworkers first enrolled the closest eligible neighboring compound adjacent to each WASH Benefits Study compound. Then they enrolled additional compounds, prioritizing those closest to WASH Benefits Study compounds, until 10 neighboring compounds were enrolled per cluster.

### Interventions

Intervention recipients in the combined WSH arm received free chlorine tablets (Aquatabs (sodium dichloroisocyanurate); Medentech, Wexford, Ireland); a safe storage vessel in which to treat and store drinking water; child potties; sanitary scoop hoes (22) to remove feces from the household; latrine upgrades to a double-pit, pour-flush latrine for all households in the compound; and handwashing stations that included detergent soap and bottles of soapy water. Local promoters visited study compounds 6 times per month, on average, during the 2-year follow-up to encourage intervention uptake. The control arm and spillover study participants did not receive interventions or health promotion.

### Procedures

Fieldworkers administered a survey to caregivers of enrolled children at the time of enrollment into the spillover study, concurrent with primary outcome measurements in the WASH Benefits Study (after 28 months of intervention). The survey was used to measure household characteristics, child illness, WSH indicators (e.g., water treatment), and neighbors’ knowledge of the WASH Benefits Study and interactions with WASH Benefits Study participants and promoters.

Due to political instability in Bangladesh, environmental and biological samples for the spillover study and the WASH Benefits Study were collected 4 months after the survey (i.e., after 32 months of intervention) to ensure safe transport and a cold chain. Fourteen children originally enrolled for the measurement of spillovers were not present to provide a stool sample; we enrolled another child in the compound aged 0–5 years to replace these children. All participants in the main trial and spillover study were offered a single dose of albendazole after stool collection regardless of infection status. Study children may have also received deworming through the national school-based deworming program. Two slides were prepared from each stool sample and analyzed using the Kato-Katz technique within 30 minutes of slide preparation (23). Laboratory technicians quantified *Ascaris lumbricoides*, hookworm, and *Trichuris trichiura* ova on each slide. Counts were double entered into a database by independent technicians. Two technicians counted 10% of the slides, and 5% were counted by a senior parasitologist for quality assurance.

In a subset of 86 control and 80 intervention clusters, fieldworkers collected drinking water samples and recorded water source (i.e., tubewell, stored water container or filter, tap). They distributed a nonporous, sterilized toy ball to each enrolled child and collected it 24 hours later. Fieldworkers hung 1.4 m of sticky fly tape at least 1.2 m from the ground near the latrine and food preparation area in a location away from smoke or stoves and protected from rain; they counted and identified the species of flies on the tape 24 hours later. Laboratory technicians enumerated *Escherichia coli* and total coliform in water samples and *E. coli* and fecal coliform in toy rinses using membrane filtration. Additional details about field procedures are in Web Appendix 1 (available at https://academic.oup.com/aje).

### Outcomes

We prespecified outcome measurement on ClinicalTrials.gov (identifier NCT02396407). We chose STH prevalence measured approximately 32 months after the intervention as the primary outcome of the spillover study because we believed spillovers were likely to affect STH transmission and because this objectively measured outcome is not subject to information bias. Stool samples with any ova were classified as positive. For each helminth, we quantified eggs per gram by multiplying the sum of egg counts from each of the duplicated slides by 12. We classified infection intensity into categories defined by the World Health Organization based on the number of eggs per gram of stool (moderate to high intensity was considered ≥5,000 eggs/g for *A. lumbricoides*, ≥1,000 eggs/g for hookworm, and ≥2,000 eggs/g for *T. trichiura*) (24).

Secondary outcomes included caregiver-reported 7-day diarrhea and respiratory illness prevalence measured approximately 28 months after the intervention. We defined diarrhea as caregiver’s report in the prior 7 days of 3 or more loose or watery stools in 24 hours or 1 or more stools with blood in 24 hours. We defined respiratory illness as caregiver’s report in the prior 7 days of persistent cough or difficulty breathing.

Although health outcomes serve as distal spillover effects, we also measured proximal spillover effects on environmental contamination after 32 months of intervention and measured WSH indicators after 28 months of intervention. Environmental contamination measures included the prevalence of *E. coli* and total coliforms in drinking water, the prevalence of *E. coli* and fecal coliforms in sentinel toy rinses, and the presence and number of synanthropic flies near the latrine and food preparation areas. WSH indicators included self-reported water treatment the day before the interview, storage of drinking water, presence of a latrine with a functional water seal, no visible feces on the latrine slab or floor, presence of a dedicated handwashing location with soap, and no visible dirt on the index child’s hands or fingernails.

### Sample size

We expected spillover effects to be smaller than effects on intervention recipients, so we powered the study to detect a relative reduction of 2.5%–6.0% in primary outcomes, which was less than the 25% relative reduction expected in the WASH Benefits Study. We assumed prevalence differences for diarrhea (change from 14.2% to 8.2%), *A. lumbricoides* (from 4.2% to 1.7%), and *T. trichiura* (from 11.2% to 7.2%), and intracluster correlation coefficients ranging from 0.023 to 0.153 based on observational studies in rural Bangladesh and India (25). Assuming 80% power and a type I error of 0.05, we calculated the required sample size for each outcome of interest, adjusting for the intracluster correlation coefficient. Given these assumptions, we planned to enroll 2,000 children in 180 clusters (90 per arm) in the spillover study.

### Statistical analyses

Two investigators (J.B.C., A.E.) independently conducted an analysis of primary and secondary outcome datasets masked to treatment assignment following a prespecified analysis protocol, which describes our analysis in full (26). Here, we provide an overview of our analysis.

Analysis was intention to treat. Because WASH Benefits Study eligibility depended on pregnancy timing, we expected trial participants and adjacent neighbors to be equivalent, on average, except for their proximity to the WSH intervention, allowing us to make inferences about spillover effects by relying only on the cluster randomization. Our primary analysis estimated unadjusted prevalence ratios and differences for binary outcomes (21) and unadjusted fecal egg-count reduction ratios (1 minus the ratio of mean intensity in intervention vs. control arm neighbors) for fecal egg counts. Our secondary analysis adjusted for covariates with bivariate associations with each outcome (likelihood ratio test *P* value < 0.2) (27). We excluded binary covariates with prevalence less than 5%. We estimated parameters using targeted maximum likelihood estimation with influence curve–based standard errors accounting for clustering (21). Analysts were masked to intervention assignment until results were replicated.

We assumed children were missing at random and conducted a complete-case analysis. For outcomes with loss to follow-up exceeding 20% of the planned sample, we used targeted maximum likelihood estimation to conduct an inverse probability of censoring-weighted analysis, which reweights measured outcomes to reconstruct the original study population as if no children had missing outcomes (28).

We assessed effect modification by the following prespecified covariates: Euclidian distance to the nearest WASH Benefits Study compound, number of steps to the nearest WASH Benefits Study compound, presence of natural physical boundaries (e.g., a pond) between spillover compounds and the nearest WASH Benefits Study compound, and the density of WASH Benefits Study compounds within a given radius of each spillover compound. All statistical analyses were completed using R, version 3.2.3 (R Foundation for Statistical Computing, Vienna, Austria).

### Human subjects protection

We received approval from the institutional review boards at the University of California, Berkeley (no. 2011-09-3652), the International Centre for Diarrheal Disease Research, Bangladesh (no. PR-11063), and Stanford University (no. 25863). Participation of human subjects did not occur until after written informed consent was obtained.

## RESULTS

**Figure 3.**
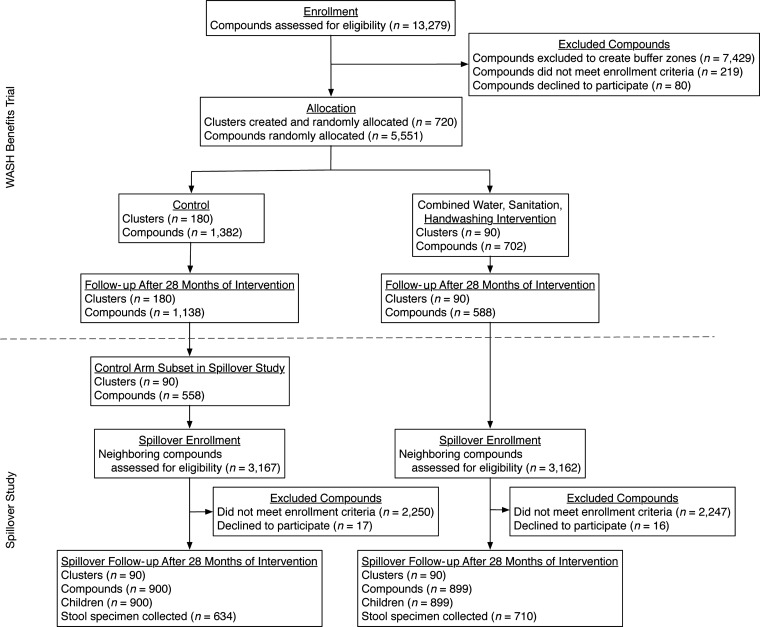
Participant flowchart, Water Quality, Sanitation, and Handwashing Benefits Study, January 2015–August 2015.

A total of 6,329 compounds neighboring WASH Benefits Study compounds were screened for eligibility in the spillover study (Figure 3). Fieldworkers enrolled 900 children in 90 control clusters (control neighbors) and 899 children in 90 WSH clusters (intervention neighbors). Overall, 75% of enrollees (*n* = 634 control neighbors and *n* = 710 intervention neighbors) provided a stool specimen.

**Figure 4.**
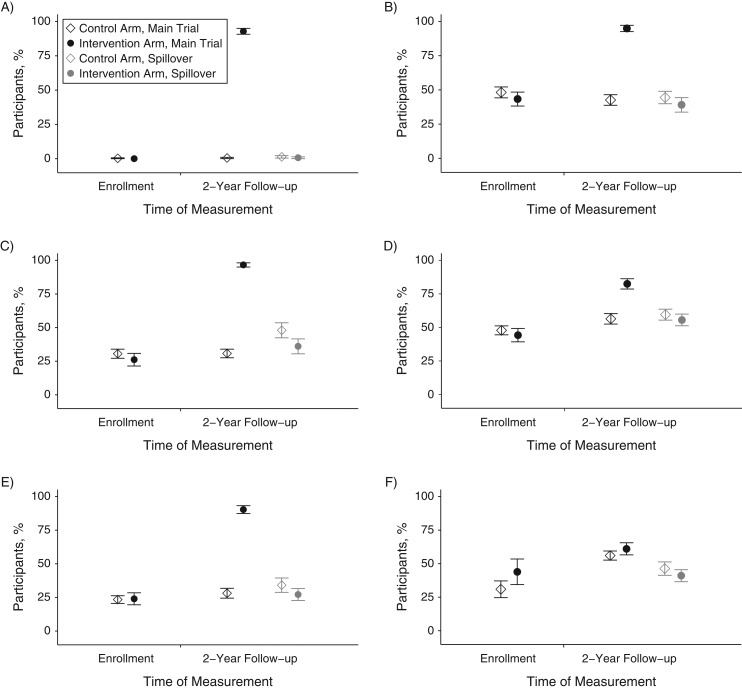
Water, sanitation, handwashing intervention uptake indicators among Water Quality, Sanitation, and Handwashing Benefits Study and spillover study participants, January 2015–August 2015. Improved water quality indicators: A) participant reported treating water yesterday or B) fieldworker observed stored drinking water in the participant’s compound. Improved sanitation indicators: fieldworker observed C) participant had access to a latrine with a functional water seal or D) no visible feces on the participant’s latrine slab or floor. Improved handwashing indicators: fieldworker observed E) a participant had a handwashing location with soap or F) no visible dirt on study child’s hands or fingernails. Circles and diamonds indicate percentage of participants. Vertical lines through each circle and diamond indicate 95% confidence intervals.

Characteristics of intervention neighbors and control neighbors enrolled in the spillover study were balanced by randomization, and neighbors’ characteristics were similar to those of WASH Benefits Study participants’ (Table 1). Self-reported deworming was balanced across arms among WASH Benefits Study participants and children in the spillover study. A total of 815 (91%) intervention neighbors and 483 (54%) control neighbors knew of the WASH Benefits Study (Web Tables 1 and 2). Among intervention neighbors, 26% had spoken with WASH Benefits Study participants and 9% had spoken with WASH Benefits Study promoters about the study. Although intervention adherence was high among WASH Benefits Study participants at follow-up, there was no evidence of intervention use among intervention neighbors, control neighbors, and WASH Benefits Study control compounds at 2-year follow-up (Figure 4).

**Table 1.** Characteristics of Participants and Nearby Neighbors by Intervention Group After 28 Months of Intervention, Water Quality, Sanitation, and Handwashing Benefits Study, Bangladesh, January 2015–August 2015

Characteristic	Neighbors of WASH Benefits Study Participants						WASH Benefits Study Participants					
	Control (*n* = 900)			Intervention (*n* = 899)			Control (*n* = 1,382)			Intervention (*n* = 702)		
	No.	%^a^	Mean (SD)	No.	%^a^	Mean (SD)	No.	%^a^	Mean (SD)	No.	%^a^	Mean (SD)
Child^b^												
Age, years			2.3 (1.1)			2.4 (1.1)			2.5 (0.2)			2.5 (0.2)
Female	391	43		439	49		419	51		239	48	
Male	509	57		460	51		404	49		257	52	
Deworming in past 6 months^c^	479	53		507	56		527	64		285	57	
Mother												
Age			26.4 (5.4)			26.4 (5.3)			25.4 (5.0)			26.1 (5.4)
Years of education			6.1 (3.5)			5.6 (3.4)			5.9 (3.5)			5.9 (3.4)
Father												
Years of education			5.2 (4.2)			4.6 (4.2)			4.9 (4.0)			5.1 (4.3)
Works in agriculture	221	25		258	29		296	21		160	23	
Household												
No. of persons per household			5.2 (1.9)			5.2 (1.9)			5.3 (2.1)			5.3 (1.9)
Has electricity	654	73		659	73		833	60		434	62	
Has a cement floor	171	19		121	13		160	12		74	11	
Acres of agricultural land owned			0.11 (0.13)			0.11 (0.17)			0.13 (0.16)			0.13 (0.16)
Meters to nearest WASH Benefits Study compound			85 (74)			70 (62)	N/A	N/A	N/A	N/A	N/A	N/A
Steps to nearest WASH Benefits Study compound			119 (107)			96 (94)	N/A	N/A	N/A	N/A	N/A	N/A
No. of WASH Benefits Study compounds within 250 m			2.7 (1.5)			2.8 (1.5)	N/A	N/A	N/A	N/A	N/A	N/A

Abbreviations: N/A, not applicable; SD, standard deviation; WASH, Water Quality, Sanitation, and Handwashing.

^a^ Some percentages were calculated using denominators that differed from the number of participants listed in column headers because of missing values for the variable of interest.

^b^ Characteristics of children in spillover study are reported in columns 2–7. Characteristics of WASH Benefits Study index children included in the soil-transmitted helminth analyses are in columns 8–13 (*n* = 823 control; *n* = 496 intervention).

^c^ Measured after 32 months of intervention, concurrent with stool specimen collection.

Median fly counts and prevalence and mean log_10_ concentrations of *E. coli* and fecal coliform in sentinel toy rinses were similar between intervention and control neighbors (Table 2). The prevalence of *E. coli* detected in drinking water was lower for intervention versus control neighbors (unadjusted prevalence ratio = 0.88; 95% confidence interval (CI): 0.80, 0.96) (Table 3). This effect was stronger among water samples collected directly from the tubewell (prevalence ratio = 0.83; 95% CI: 0.73, 0.95), and there was no effect among samples from stored drinking water (prevalence ratio = 1.02; 95% CI: 0.95, 1.10). The prevalence of total coliforms was similar between arms in all drinking water samples regardless of water source.

**Table 2.** Synanthropic Fly^a^ Counts and Ratios of Fly Counts Between Intervention Arms Among Neighbors After 32 Months Intervention, Water Quality, Sanitation, and Handwashing Benefits Study, Bangladesh, January 2015–August 2015

Fly Capture Location	Control Neighbors				Intervention Neighbors				Unadjusted Ratio of Fly Counts	95% CI^b^
	No. of Compounds	Median (SD) Fly Count	No. With Any Flies	% With Any Flies	No. of Compounds	Median (SD) Fly Count	No. With Any Flies	% With Any Flies		
Near latrine	708	3 (13)	553	78	703	3 (21)	576	82	1.16	0.81, 1.66
Near cooking area	718	3 (23)	570	79	711	3 (21)	559	79	0.88	0.64, 1.21

Abbreviations: CI, confidence interval; SD, standard deviation.

^a^ Includes *Musca domestica*, bottle flies (Calliphoridae), flesh fly (Sarcophagidae), lesser house fly (*Fannia canicularis*).

^b^ Standard errors account for clustering at the study cluster level.

**Table 3.** Sentinel Toy and Drinking Water Contamination Among Neighbors After 32 Months Intervention, Water Quality, Sanitation, and Handwashing Benefits Study, Bangladesh, January 2015–August 2015

Measurement	No. of Compounds	Mean log_10_ CFU/100 mL^a^ (SD)	No. of Positive Samples	% Positive Samples	No. of Compounds	Mean log_10_ CFU/100 mL^a^ (SD)	No. of Positive Samples	% Positive Samples	Unadjusted Prevalence Ratio	95% CI^b^
Sentinel toys										
*Escherichia coli*	700	1.5 (1.3)	558	80	697	1.5 (1.3)	581	83	1.05	0.99, 1.11
Fecal coliforms	700	3.4 (1.1)	695	99	697	3.2 (1.2)	691	99	1.00	0.99, 1.01
Drinking water										
*Escherichia coli*										
All samples^c^	718	0.9 (0.9)	553	77	713	0.7 (1.0)	481	67	0.88	0.80, 0.96
Samples from tubewell	424	0.6 (0.9)	281	66	470	0.4 (0.8)	259	55	0.83	0.73, 0.95
Samples from stored water	258	1.3 (0.8)	238	92	219	1.5 (0.8)	206	94	1.02	0.95, 1.10
Total coliforms										
All samples^c^	718	2.1 (0.5)	710	99	713	2.0 (0.6)	700	98	0.99	0.98, 1.01
Samples from tubewell	424	1.9 (0.6)	416	98	470	1.8 (0.7)	457	97	0.99	0.97, 1.01
Samples from stored water	258	2.3 (0.2)	258	100	219	2.3 (0.1)	219	100	–^d^	–^d^

Abbreviations: CFU, colony-forming unit; CI, confidence interval; SD, standard deviation.

^a^ For values below the detection limit (1 CFU/100 mL for water, 2.5 CFU/100 mL for toy rinses), we imputed 0.5 prior to taking the logarithm.

^b^ Standard errors account for clustering at the study cluster level.

^c^ Includes 55 compounds in which residents drew drinking water samples directly from a piped water source; these were not included in a separate stratification category, because of the low number of observations. A total of 903 drinking water samples (63%) provided by participants were collected from tubewells, 487 (33%) were from stored water, 55 (4%) were from piped water, and 3 (<1%) were from water filters.

^d^ Prevalence ratio could not be estimated because all samples contained total coliforms.

**Figure 5.**
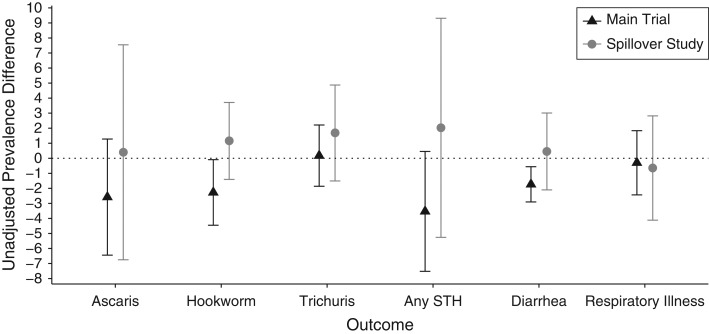
Unadjusted prevalence differences for the intervention versus control arms between intervention recipients and their neighbors, Water Quality, Sanitation, and Handwashing Benefits Study, January 2015–August 2015. In the main trial, soil-transmitted helminth infection was measured among index children, preschool age children, and school-aged children; diarrhea was measured among children younger than 36 months in the compound at enrollment; and respiratory illness was measured among index children and all other children younger than 5 years in the compound 2 years after the intervention. In the spillover study, all health outcomes were measured among children 0–5 years of age. Circles and triangles indicate unadjusted prevalence differences. Vertical lines through each circle and triangle indicate 95% confidence intervals.

Among control neighbors, the prevalence of *A. lumbricoides* was 31.4%, of hookworm was 3.6%, and of *T. trichiura* was 3.9% (Table 4). There were no differences in STH prevalence comparing intervention and control neighbors: *A. lumbricoides* prevalence difference (PD) = 0.00 (95% CI: −0.07, 0.08); hookworm PD = 0.01 (95% CI: −0.01, 0.04); *Trichuris* PD = 0.02 (95% CI: −0.02, 0.05); and any STH infection PD = 0.02 (95% CI: −0.05, 0.09) (Table 4, Figure 5). There were also no reductions in geometric fecal egg counts (Table 5). Prevalence of moderate or heavy infections was less than 5% for all helminths among intervention and control neighbors (Web Table 3). Four percent of control neighbors (*n* = 634) and 5% of intervention neighbors (*n* = 711) were infected with more than 1 helminth. The prevalence was also similar among intervention neighbors and control neighbors for diarrhea (8.0% vs. 7.6%) and respiratory illness (8.6% vs. 9.2%) (Table 4).

**Table 4.** Prevalence and Unadjusted Prevalence Ratios and Differences for Diarrhea, Respiratory Illness, and Soil-Transmitted Helminth Infection Among Children Neighboring Compounds After 32 Months of Intervention, Water Quality, Sanitation, and Handwashing Benefits Study, Bangladesh, January 2015–August 2015

Outcome	Control Neighbors		Intervention Neighbors		Unadjusted Prevalence Ratio^a^	95% CI^b^	Unadjusted Prevalence Difference^a^	95% CI^b^
	No.	%	No.	%				
Diarrhea	898	7.6	897	8.0	1.06	0.76, 1.47	0.00	−0.02, 0.03
Respiratory illness	898	9.2	897	8.6	0.93	0.63, 1.37	−0.01	−0.04, 0.03
Soil-transmitted helminth								
*Ascaris lumbricoides*	634	31.4	711	31.8	1.01	0.81, 1.27	0.00	−0.07, 0.08
Hookworm	634	3.6	711	4.8	1.32	0.72, 2.42	0.01	−0.01, 0.04
*Trichuris trichiura*	634	3.9	711	5.6	1.43	0.75, 2.72	0.02	−0.02, 0.05
Any soil-transmitted helminth	634	34.5	711	36.6	1.06	0.86, 1.30	0.02	−0.05, 0.09

Abbreviation: CI, confidence interval.

^a^ Prevalence ratios and differences compare the prevalence among intervention neighbors with the prevalence among control neighbors.

^b^ Standard errors account for clustering at the study cluster level.

**Table 5.** Soil-Transmitted Helminth Infection Intensity Among Children Neighboring Study Compounds After 32 Months of Intervention, Water Quality, Sanitation, and Handwashing Benefits Study, Bangladesh, January 2015–August 2015

Soil-Transmitted Helminth	Control Neighbors		Intervention Neighbors		Fecal Egg-Count Reduction Ratio^a^	95% CI^b^	Mean Fecal Egg-Count Difference	95% CI^b^
	No.	Geometric Mean	No.	Geometric Mean				
*Ascaris lumbricoides*	634	3.23	711	3.92	0.16	−0.27, 0.60	0.00	−0.92, 0.93
Hookworm	634	0.21	711	0.24	0.02	−0.11, 0.16	−0.48	−1.05, 0.10
*Trichuris trichiura*	634	0.2	711	0.32	0.10	−0.09, 0.30	2.44	−2.34, 7.21

Abbreviation: CI, confidence interval.

^a^ Fecal egg-count reduction ratio: (1 − RR) × 100%, where the RR is the ratio of mean eggs per gram in the intervention arm versus the control arm.

^b^ Standard errors account for clustering at the study cluster level.

Adjusted and inverse probability of censoring-weighted analyses produced similar results (Web Table 4). For all outcomes, prevalence ratios and differences comparing neighbors of intervention participants with neighbors of control participants were similar across levels of effect modifiers (Web Figures 1–7).

## DISCUSSION

We measured spillover effects of a combined WSH intervention on environmental contamination, hygienic behavior, and infectious disease outcomes in young children. By enrolling neighbors of randomly allocated trial participants, we were able to estimate geographically local spillover effects while relying on the original trial’s randomization for inference. We hypothesized that spillovers would occur through 3 possible mechanisms: 1) reduced environmental fecal contamination; 2) reduced pathogen transmission from intervention recipients to neighbors, resulting from recipients’ lower disease burden due to intervention; or 3) behavior change among neighbors. We found evidence of spillovers through the environmental mechanism: Neighbors of intervention recipients were less likely to have *E. coli* detected in their tubewell water. However, there was no evidence of reductions in other measures of environmental contamination or of STH infection, diarrhea, or respiratory illness among intervention neighbors compared with control neighbors.

For our environmental assessment, we measured proximal spillover effects on environmental contamination. We found lower *E. coli* concentration in tubewells of intervention neighbors compared with those of control neighbors. Though we did not find reductions in total coliforms in tubewell water, this indicator includes bacteria not of fecal origin (29) and is less sensitive to changes in fecal input into the environment than is *E. coli*. Improvements in latrine infrastructure may have reduced leakage into the groundwater (30); researchers have found fecal indicator bacteria in groundwater up to 2 m from pit latrines and up to 24.5 m in sandy soil (31). We did not find reductions in environmental contamination as measured by fly density, sentinel toys, and stored water, which capture surface level contamination. Together, these findings suggest possible spillover effects through groundwater but not surface level environmental contamination. Secondary contamination through poor hand hygiene, for example, may have counteracted improvements to source water quality.

Spillover effects may also have occurred if neighbors adopted interventions, but we found no evidence of intervention or behavior adoption. Limited hardware availability and lack of resources to purchase hardware likely inhibited diffusion of interventions to neighbors. Dual-pit latrines would have been costly for neighbors to construct themselves, and Aquatabs and the water storage container delivered as part of the WASH Benefits Study were not sold locally. Spillover effects may have been more likely if neighbors discussed interventions with WASH Benefits Study participants or saw them in use; however, only 26% of neighbors reported discussing the WASH Benefits Study with intervention recipients. Finally, the absence of behavior change among neighbors may reflect limited knowledge of or perceived harm of illness or low social desirability of the WASH Benefits Study interventions (32).

There are several features of this study that limit the generalizability of our findings. First, the intervention was only delivered to approximately 10% of each cluster on average. From the baseline survey of WASH Benefits Study households, which was fairly representative of study clusters as a whole, intervention coverage was approximately 30% for the water and handwashing components and 20% for the sanitation component, as measured by indicators shown in Figure 4. Thus, by 2-year follow-up, when we measured spillover effects, overall intervention coverage in study clusters was likely to be less than 50%. WSH interventions delivered to entire populations (e.g., introduction of municipal piped water and sewerage) have been reported to be associated with reduced enteric infection (14–17). It is possible that a higher level of intervention coverage must be reached for WSH interventions to yield spillover effects. This is true for vaccines, many of which confer benefits to nonrecipients once immunization coverage reaches a herd immunity threshold (typically >75%) (7). Some vaccines provide indirect protection to unvaccinated individuals at coverage levels below the herd immunity threshold.

Second, in the original WASH Benefits Study, the combined WSH intervention led to small reductions in STH prevalence (PD = −3.5%; 95% CI: −7.5, 0.5) and diarrhea (PD = −1.7%; 95% CI: −2.9, −0.6) (20). The size of spillover effects may be correlated with the size of effects on intervention recipients (33); for example, a large reduction in environmental contamination among intervention recipients would be more likely to translate into large spillover effects for neighbors than would a small reduction for intervention recipients. However, in this study, impacts on intervention recipients’ health and environmental contamination may have been too modest to reduce transmission to neighbors.

Our study is subject to several limitations. First, we measured diarrhea and respiratory illness through caregiver report. Poor recall may have led to misclassification; however, because neighbors did not receive interventions, any misclassification was likely to be nondifferential by study arm, which would have biased results toward the null. Second, the double-slide Kato-Katz technique has low sensitivity in low-infection intensity settings such as Bangladesh, where large-scale, school-based deworming programs have been offered since 2008 (34). This may have limited our statistical power to detect spillover effects, which we would expect to be smaller than effects on intervention recipients. Finally, we did not define social networks. There is some evidence that enteric and respiratory pathogens can spread through social networks (35); although this has been examined for WSH interventions in few studies, spillovers through social networks are theoretically plausible (18).

### Conclusion

A compound-level, combined WSH intervention reduced contamination of neighbors’ tubewell water but did not lead to spillovers for other proximal measures of contamination in the domestic environment or for child health outcomes. For proximal spillover effects to translate to distal spillover effects, improvements in neighbors’ health behaviors may have been necessary. Alternatively, spillover effects may be more pronounced in populations with higher disease transmission or higher levels of WSH intervention coverage in the community.

## Supplementary Material

Web Material

## Abbreviations

CI: confidence interval
PD: prevalence difference
STH: soil-transmitted helminths
WASH: Water Quality, Sanitation, and Handwashing
WSH: water, sanitation, and handwashing
