# Supplementary material for: A Randomized Controlled Trial to Measure Spillover Effects of a Combined Water, Sanitation, and Handwashing Intervention in Rural Bangladesh
Source: Am J Epidemiol. 2018 Mar 27;187(8):1733–44. doi: 10.1093/aje/kwy046 (PMC6070113; doi:10.1093/aje/kwy046)

Web Appendix 1

Description of field procedures

Water and toy samples

Fieldworkers asked respondents to bring a glass of water that they would give to their young children and asked them to pour the water directly into a sterile Whirlpak bag (Nasco Modesto, Salida, CA) without touching the cup to the bag. They distributed distributed toy balls to study children in a subset of 86 control clusters and 80 intervention clusters. Fieldworkers placed the ball in a sterile Whirlpak bag with 250 ml of distilled water, massaged it from outside the bag for 15 seconds, and shook the bag for 15 seconds.

Water and toy rinse samples were transported on ice to the laboratory and analysed within 12 hours of collection. Laboratory technicians enumerated E. coli and total coliform in water samples and E. coli and faecal coliform in toy rinses using membrane filtration. They filtered 100 ml of each sample through a cellulose filter with 0.45 micrometer sized pores; serial dilutions (1:10, 1:100) were performed for toy rinse samples. They incubated water samples at 35°C and toy rinse samples at 44.5° C on MI Agar plates for 24 hours. 10% field blanks and one laboratory control per sample processor per day were analysed for quality control. For 5% of samples, replicate aliquots from the same Whirlpak bag were processed. Trained technicians counted colonies on plates immediately following incubation and expressed counts in colony forming units (CFU) per 100 mL of water and per toy. Plates with 300 or more colonies were classified as too numerous to count.

Fly density

Fieldworkers hung 4.5 feet of sticky fly tape at least four feet from the ground near the latrine and food preparation area in a location away from any smoke or stoves and protected from rain (e.g., under a roof) if possible. They counted the number of each of the following species that were stuck to the tape: house flies (*Musca domestica*), flesh flies (*Sarcophaga spp.*), bottle flies (predominantly *Calliphora spp., Lucilia spp., Chrysomya spp.*) and lesser house flies (*Fannia canicularis*). They did not count non-synanthropic insects, such as fruit flies and wasps.

Stool collection

Fieldworkers provided caregivers with plastic stool collection containers and returned the following morning between 8am and 10am to collect stool samples from that morning's defecation. Fieldworkers aliquoted 2 ml of fresh stool into sterile containers, barcoded each container with a unique identification number, and transported samples to the laboratory.

Web Table 1: Neighbors of WASH Benefits participants' exposure to the WASH Benefits participants and interventions

	Control Neighbors N (%)	Intervention neighbors N (%)
Know of WASH Benefits activities in their cluster	483 (54%)	815 (91%)
Could name the WASH Benefits study	4 (0.4%)	4 (0.4%)
Could name WASH Benefits interventions		
Handwashing station (stool, bowl, soapy water bottle)	0 (0%)	779 (87%)
Soap	0 (0%)	92 (10%)
Sani scoop	1 (0%)	437 (49%)
New latrine	0 (0%)	518 (58%)
Improved latrine	2 (0%)	203 (23%)
Child potty	0 (0%)	507 (56%)
Aquatab	1 (0%)	172 (19%)
Water storage vessel	0 (0%)	458 (51%)
Reported talking to WASH Benefits participants about the study	14 (2%)	230 (26%)
Reported talking to a WASH Benefits promoter about the study	2 (0%)	83 (9%)

Web Table 2: Topics neighbors discussed with WASH Benefits participants and community health promoters

	Discussed with WASH Benefits Participants		Discussed with WASH Benefits Community Health Promoter	
	Control neighbors (%)	Intervention neighbors (%)	Control neighbors (%)	Intervention neighbors (%)
Handwashing in general	2 (0%)	6 (1%)	0 (0%)	7 (1%)
Handwashing with soap	5 (1%)	144 (16%)	0 (0%)	63 (7%)
Handwashing with soapy water bottle	0 (0%)	80 (9%)	0 (0%)	25 (3%)
Using a handwashing station	0 (0%)	56 (6%)	0 (0%)	14 (2%)
Making a handwashing station	0 (0%)	9 (1%)	0 (0%)	2 (0%)
Using an improved latrine	5 (1%)	40 (5%)	0 (0%)	19 (2%)
Improving one's latrine	0 (0%)	15 (2%)	0 (0%)	7 (1%)
Cleaning one's latrine regularly	0 (0%)	44 (5%)	0 (0%)	25 (3%)
Emptying the latrine pit	0 (0%)	0 (0%)	0 (0%)	1 (0%)
Using a sani scoop	0 (0%)	45 (5%)	0 (0%)	11 (1%)
Using a child potty	1 (0%)	60 (7%)	0 (0%)	23 (3%)
Safe storage of water	0 (0%)	76 (9%)	0 (0%)	18 (2%)
Using Aquatabs	0 (0%)	20 (2%)	0 (0%)	3 (0%)
Taking deworming medication	1 (0%)	1 (0%)	0 (0%)	0 (0%)
Free deworming medication at school	0 (0%)	1 (0%)	0 (0%)	0 (0%)

Web Table 3: Prevalence of moderate or heavy soil-transmitted helminth infection after 32 months of intervention among neighbors of WASH Benefits participants by intervention arm

	Control neighbors (N=634)	Intervention neighbors (N=711)
	Prevalence (95% CI)[a]	Prevalence (95% CI)
Ascaris lumbricoides	3.2 (1.6, 4.7)	4.5 (2.7, 6.3)
Hookworm	0.2 (-0.2, 0.5)	0.0 (–,–)[b]
Trichuris trichiura	0.2 (-0.2, 0.5)	0.4 (-0.2, 1.0)
Any soil-transmitted helminth	3.3 (1.7, 4.9)	4.9 (2.9, 6.9)

[a] Standard errors account for clustering at the study cluster level.
[a] Standard errors could not be estimated because there were no moderate or heavy infections.

Web Table 4: Adjusted and inverse probability of censoring weighted prevalence ratios and differences for soil-transmitted helminth infection among children neighboring WASH Benefits compounds after 32 months of intervention (N=1,345)

	Adjusted prevalence ratio[a] (95% CI)[b]	IPCW[b] prevalence ratio (95% CI)	Adjusted prevalence difference[a] (95% CI)	IPCW prevalence difference (95% CI)
**Soil-transmitted helminth prevalence**				
Ascaris lumbricoides	0.95 (0.80, 1.14)	0.96 (0.80, 1.15)	-0.02 (-0.07, 0.04)	-0.01 (-0.07, 0.04)
Hookworm	1.41 (0.80, 2.48)	1.43 (0.83, 2.44)	0.01 (-0.01, 0.04)	0.01 (-0.01, 0.04)
Trichuris trichiura	1.18 (0.67, 2.07)	1.24 (0.73, 2.11)	0.01 (-0.02, 0.04)	0.01 (-0.02, 0.04)
Any soil-transmitted helminth	1.01 (0.85, 1.19)	1.01 (0.86, 1.19)	0.00 (-0.06, 0.06)	0.00 (-0.05, 0.06)
	Adjusted geometric FECR[d] (95% CI)[b]	IPCW[c] geometric FECR (95% CI)	Adjusted arithmetic FECR (95% CI)	IPCW arithmetic FECR (95% CI)
**Soil-transmitted helminth infection eggs per gram**				
Ascaris lumbricoides	-0.01 (-0.31, 0.28)	0.01 (-0.28, 0.30)	-0.17 (-0.83, 0.50)	-0.16 (-0.78, 0.45)
Hookworm	0.03 (-0.09, 0.15)	0.03 (-0.08, 0.14)	-0.51 (-1.02, -0.01)	-0.49 (-0.98, -0.01)
Trichuris trichiura	0.05 (-0.11, 0.20)	0.06 (-0.09, 0.20)	2.12 (-2.10, 6.34)	2.26 (-2.22, 6.74)

[a] Prevalence ratios and differences compare the prevalence among intervention neighbors to the prevalence in control neighbors.
[b] Standard errors account for clustering at the study cluster level.
[c] Inverse probability of censoring weighted estimate.
[d] Fecal egg count reduction ratio: (1-RR) x 100%, where the RR is the ratio of mean eggs per gram in the intervention vs. control arm.

Web Figure 1: Unadjusted prevalence differences for STH infection among children neighboring WASH Benefits compounds after 32 months of WASH Benefits intervention stratified by potential effect modifiers

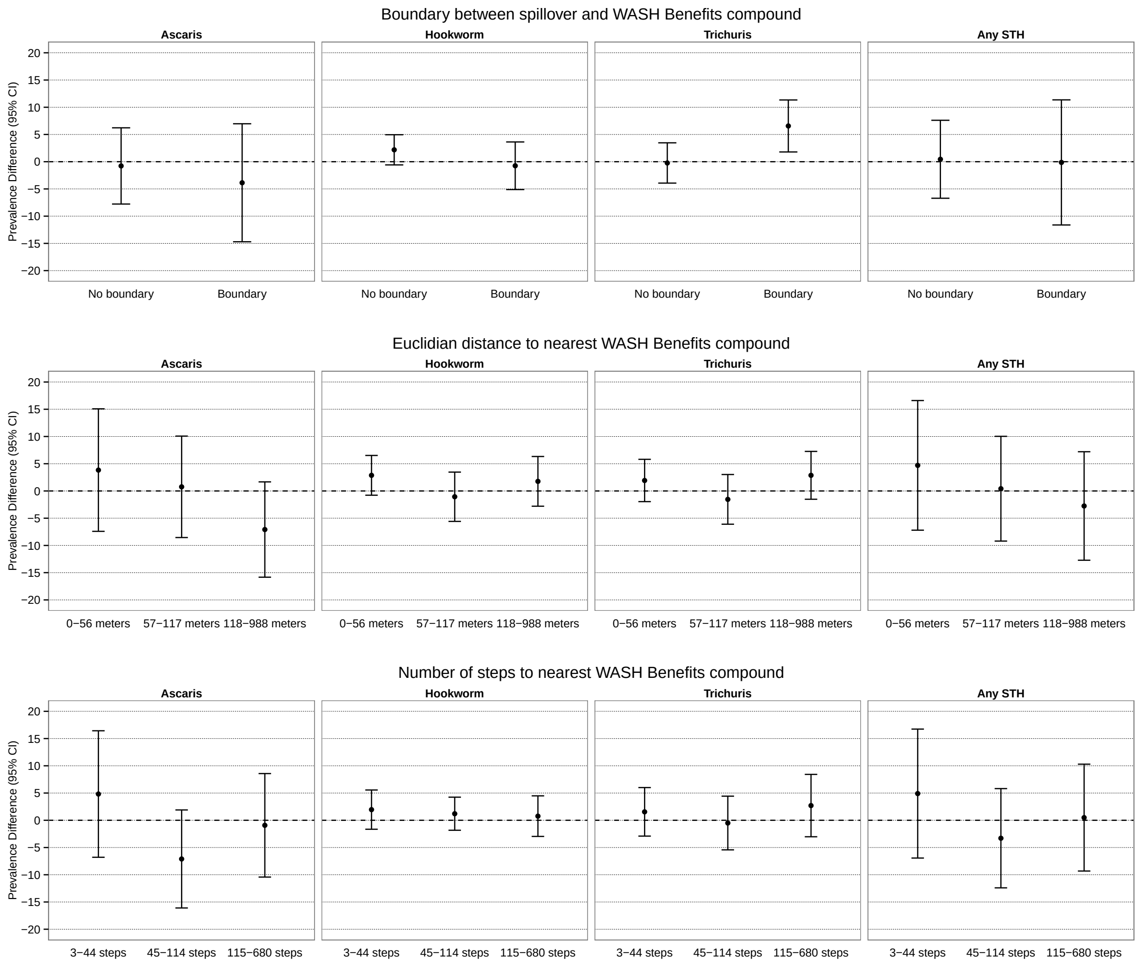

Web Figure 2: Unadjusted faecal egg count reduction among children neighboring WASH Benefits compounds after 32 months of WASH Benefits intervention stratified by potential effect modifiers

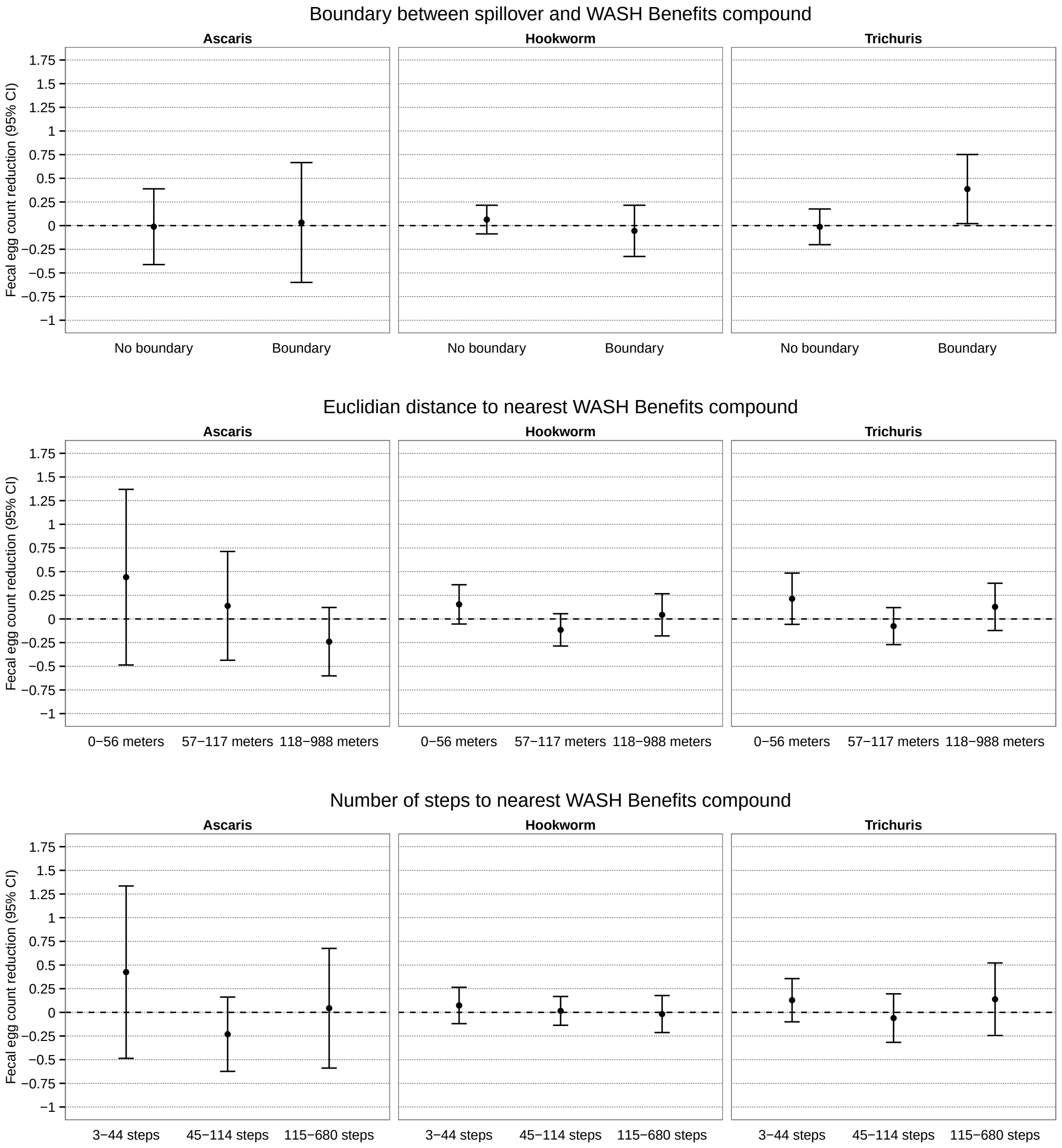

Web Figure 3: Unadjusted prevalence differences for diarrhoea and respiratory illness among children neighboring WASH Benefits compounds after 32 months of WASH Benefits intervention stratified by potential effect modifiers

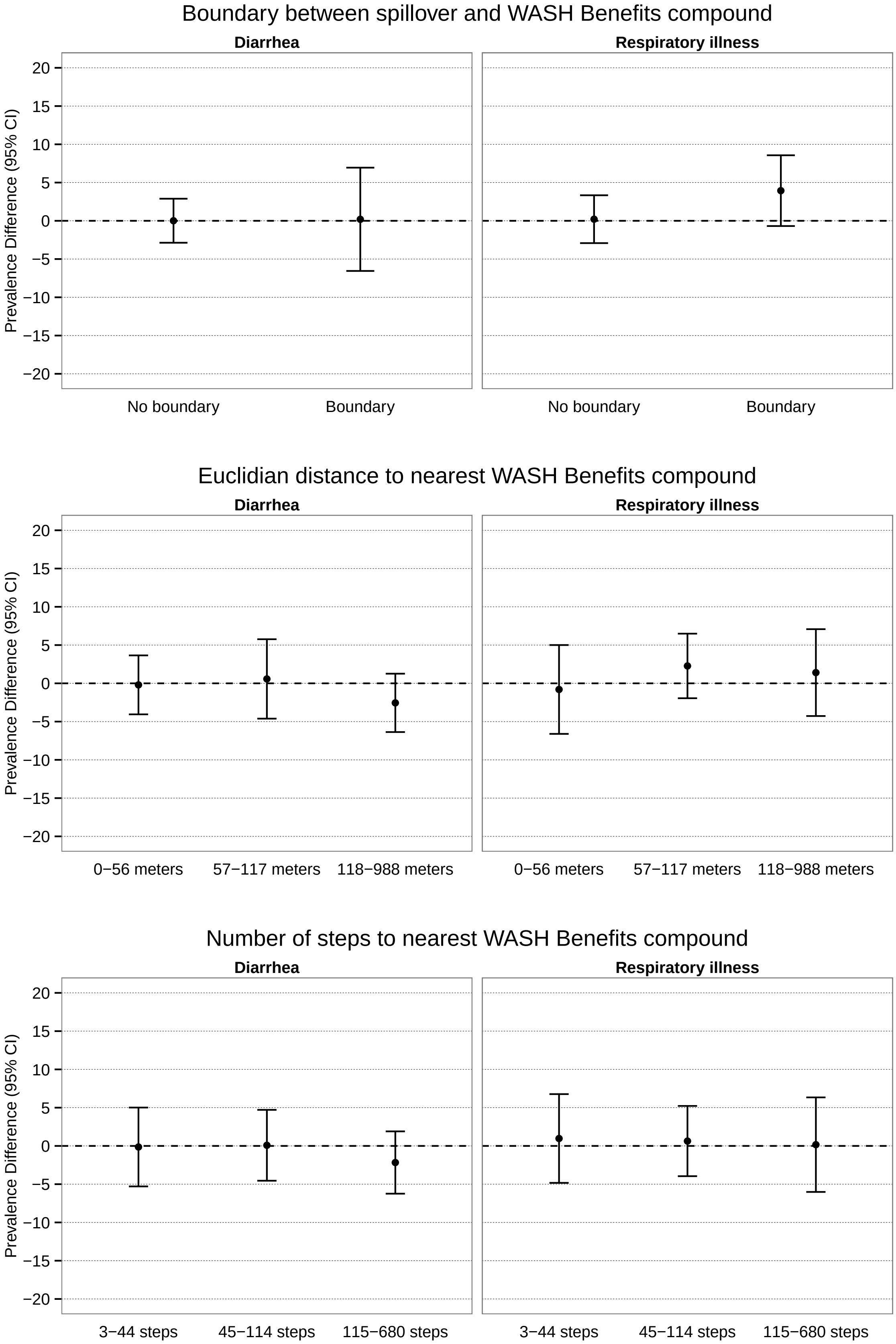

Web Figure 4: Unadjusted prevalence differences for STH infection among children neighboring WASH Benefits compounds after 32 months of WASH Benefits intervention stratified by treatment density

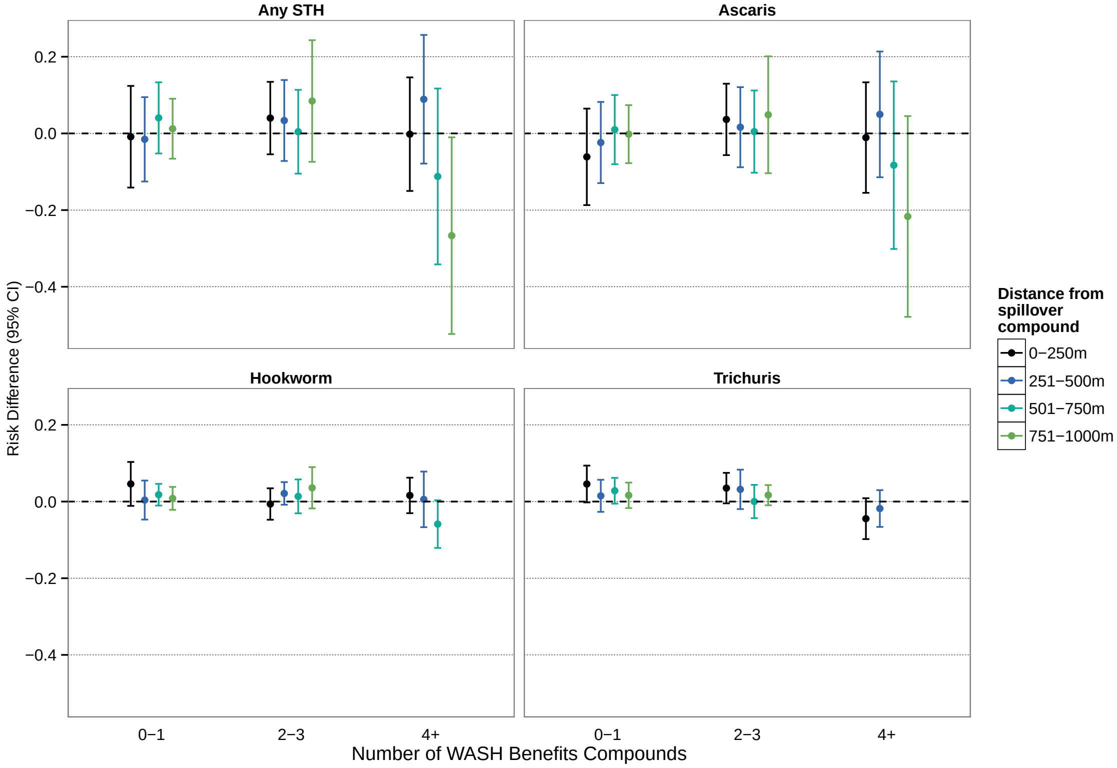

Web Figure 5: Unadjusted faecal egg count reduction among children neighboring WASH Benefits compounds after 32 months of WASH Benefits intervention stratified by treatment density

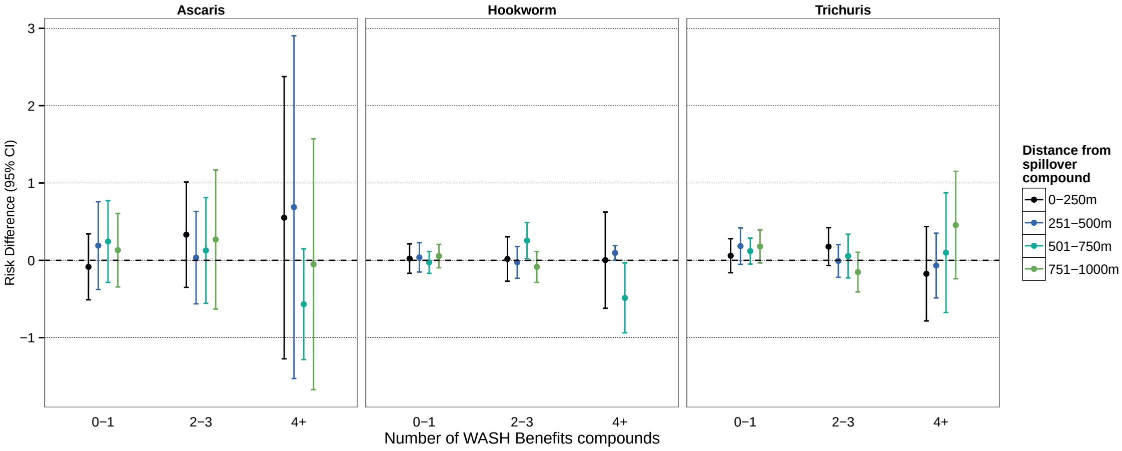

Web Figure 6: Unadjusted prevalence differences for diarrhoea among children neighboring WASH Benefits compounds after 32 months of WASH Benefits intervention stratified by treatment density

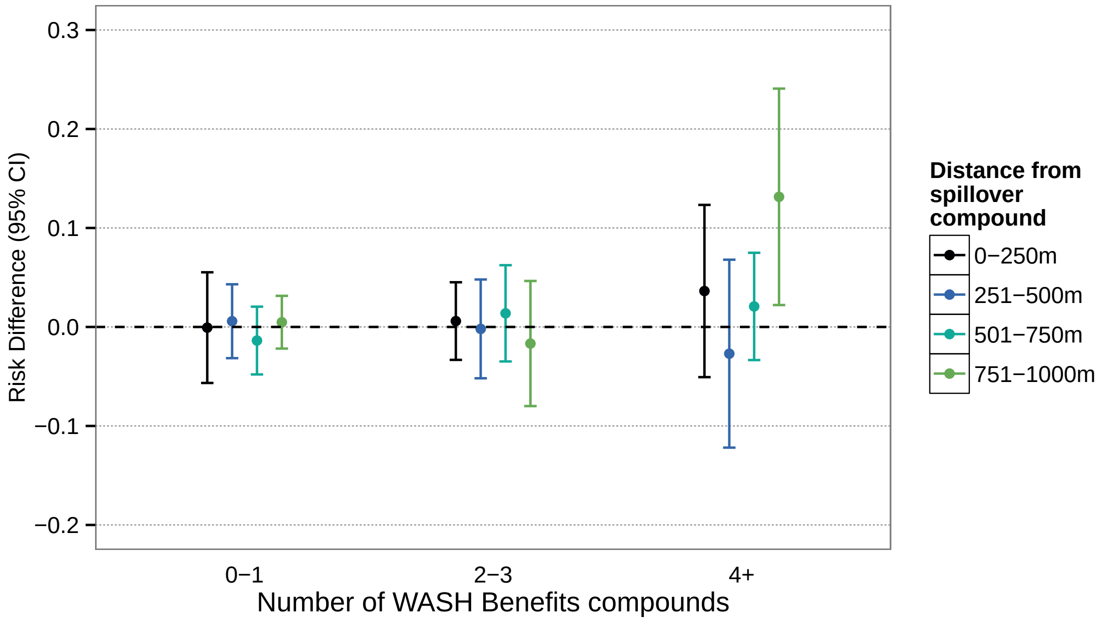

Web Figure 7: Unadjusted prevalence differences for respiratory illness among children neighboring WASH Benefits compounds after 32 months of WASH Benefits intervention stratified by treatment density

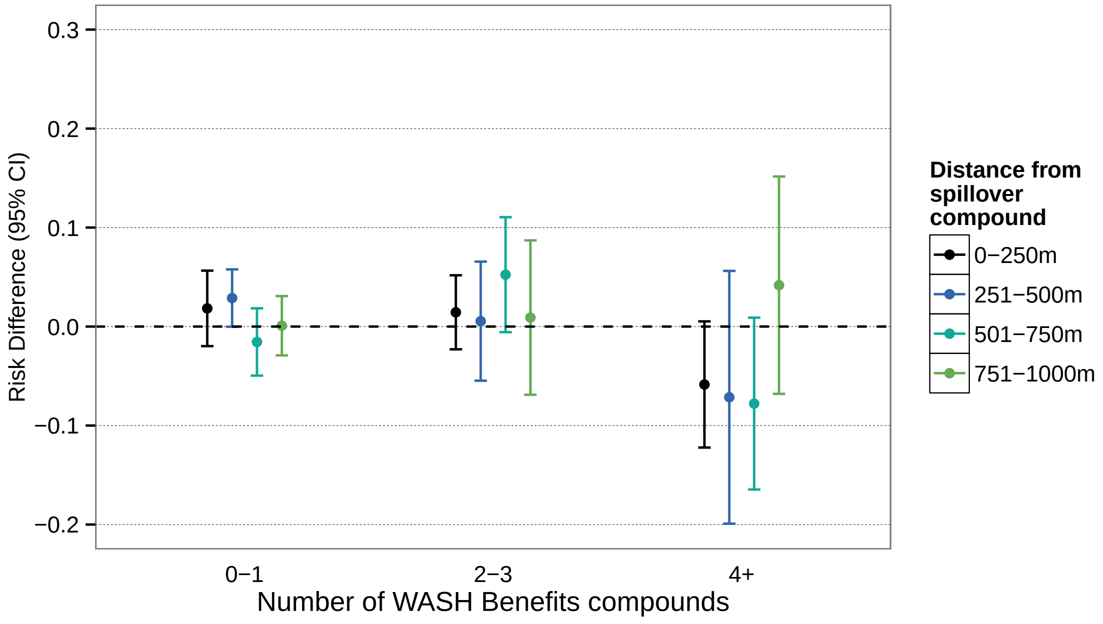

Supplement: Web Material [file kwy046_benjamin-chung_web_material_final.pdf]
